# Active Estrogen–Succinate Metabolism Promotes Heme Accumulation and Increases the Proliferative and Invasive Potential of Endometrial Cancer Cells

**DOI:** 10.3390/biom13071097

**Published:** 2023-07-10

**Authors:** Jia-Jing Lu, Xing Zhang, Ayitila Abudukeyoumu, Zhen-Zhen Lai, Ding-Yu Hou, Jiang-Nan Wu, Xiang Tao, Ming-Qing Li, Xiao-Yong Zhu, Feng Xie

**Affiliations:** 1 Medical Center of Diagnosis and Treatment for Cervical and Intrauterine Diseases, Obstetrics and Gynecology Hospital of Fudan University, Shanghai 200011, China; 2 Laboratory for Reproductive Immunology, Hospital of Obstetrics and Gynecology, Shanghai Medical School, Fudan University, Shanghai 200080, China; 3 Department of Gynecology, Shanghai Jiading Maternal Child Health Hospital, Shanghai 201800, China; 4 Department of Gynecology, Hospital of Obstetrics and Gynecology, Shanghai Medical School, Fudan University, Shanghai 200011, China; 5 Clinical Epidemiology, Clinical Research Center, Obstetrics and Gynecology Hospital of Fudan University, Shanghai 200080, China; 6 Department of Pathology, Hospital of Obstetrics and Gynecology, Shanghai Medical School, Fudan University, Shanghai 200011, China; 7 Shanghai Key Laboratory of Female Reproductive Endocrine Related Diseases, Hospital of Obstetrics and Gynecology, Fudan University, Shanghai 200080, China

**Keywords:** uterine endometrial cancer, estrogen, succinate, heme, NCOA1

## Abstract

Uterine endometrial cancer (UEC) is an estrogen-related tumor. Succinate and heme metabolism play important roles in the progression of multiple tumors. However, the relationship between estrogen, succinate, and heme metabolism and related regulatory mechanisms remain largely unknown. In this study, we observed that the expression of aminolevulinate delta synthase 1 (ALAS1) and solute carrier family member 38 (SLC25A38) in UEC tissues is significantly higher than that in normal tissues. Further analysis showed that estrogen and succinate increased the expression of ALAS1 and SLC25A38 in uterine endometrial cancer cells (UECC), and the administration of succinate upregulated the level of the estrogen receptor (ER). Silencing nuclear receptor coactivator 1 (NCOA1) reversed the effects of estrogen and succinate via downregulation of ALAS1 expression. Additionally, exposure of UECC to heme increased cell viability and invasiveness, while silencing the NCOA1 gene weakened this effect. These findings revealed that estrogen and succinate can synergistically increase the expression of ALAS1 and SLC25A38 via the ERβ/NCOA1 axis, promoting heme accumulation and increasing the proliferative and invasive potential of UECC.

## 1. Introduction

Uterine endometrial cancer (UEC) is one of the most common malignancies for females, accounting for 30% of malignant tumors in the female reproductive system. Its incidence has gradually increased in recent years and has shown a trend of rejuvenation [1,2]. By the year 2030, the incidence of UEC is expected to reach 42.13/100,000 women, which is an increase of nearly 55% compared with that of 2010 [3]. According to the Bokhman classification, UEC can be divided into two types [4]. Type I is estrogen-dependent, where in the presence of chronic exposure to estrogen unopposed by progesterone, the endometrium undergoes a progression of simple hyperplasia, complex hyperplasia, atypical hyperplasia, and even carcinogenesis [5]. Type II is non-estrogen-dependent, and its pathogenesis is not clearly related to estrogen. Serous carcinoma, clear cell carcinoma, and some other rare, high-grade tumors belong to this type, and patients usually have poor prognoses [4,6,7].

Type I UEC accounts for 70–80% of all UEC cases. Among them, grade I/II endometrioid carcinoma is the most common histological type. Patients often present with concomitant obesity, hypertension, or diabetes or have a history of taking hormonal drugs such as tamoxifen [5]. It has been reported that by upregulating the c-MYC gene, estrogen upregulates the level of glutaminase in estrogen-sensitive uterine endometrial cancer cells (UECC), increasing cell vitality and decreasing autophagy [8]. Abnormal glycolipid metabolism is closely related to the occurrence of UEC [9,10]. The tricarboxylic acid cycle (TCA cycle) is the main way for the body to obtain energy, and it is the final metabolic pathway of the three major nutrients (protein, carbohydrates, and fats) [11,12]. Succinate dehydrogenase B (SDHB) is a metabolic enzyme in the TCA cycle [13]. Many studies have shown that germline mutations of SDHB can cause the degradation of the SDHB protein, promoting tumorigenesis and the abnormal accumulation of succinate [11,12]. Our previous study proved that estrogen can inhibit the expression of SDHB through the PPARγ-SP1-UBC pathway, leading to the accumulation of succinate and accelerating the progression of UEC [14].

Heme has regulatory effects on cellular metabolism and is of great physiological significance in maintaining cellular homeostasis [15]. Aminolevulinate delta synthase 1 (ALAS1) is the key enzyme in heme biosynthesis and requires pyridoxal phosphate as a cofactor [16]. In the TCA cycle, α-ketoglutaric acid produces succinyl CoA under the action of dehydrogenase, and succinate can also be converted to succinyl CoA [17]. Glycine can be transported into mitochondria through the solute carrier family member 38 (SLC25A38) and bind to succinyl CoA via ALAS1. After a series of complex reactions, finally, heme is generated [18,19]. Mediated by heme oxygenase 1 (HMOX1), free heme can be degraded into iron, carbon monoxide, and biliverdin [19,20]. Our recent study showed that in patients with endometrial hyperplasia complicated by abnormal uterine bleeding, heme metabolism plays a regulatory role in the abnormal infiltration of immune cells [21]. However, the characteristics and mechanism of estrogen–succinate–heme metabolism in UEC has not yet been clarified.

In this study, we focused on heme metabolism in the microenvironment of UEC. We explored the characteristics of estrogen–succinate–heme metabolism, aiming to provide a theoretical basis and potential target for the anticancer treatment of UEC.

## 2. Materials and Methods

### 2.1. Patients and Sample Collection

All patients had undergone curettage or hysteroscopy previously and were confirmed pathologically as having grade I/II endometrioid carcinoma after receiving total hysterectomies. We ruled out participants who had other immune diseases, tumors, or a history of hormone drug use. The UEC tissues and paired adjacent healthy tissues were obtained from the same patients (Table 1). The protocol for the present study was approved by the Human Research Ethics Committee of the Obstetrics and Gynecology Hospital, Fudan University (Shanghai, China), and written informed consent was obtained from all participants.

### 2.2. Cell Lines

The human uterine endometrial cancer cells (UECC) Ishikawa and RL95-2 were obtained from the Type Culture Collection of the Chinese Academy of Sciences (Shanghai, China), and both of them were cultured in DMEM/F12 medium (HyClone, Logan, UT, USA) supplemented with 10% fetal bovine serum (FBS; Gibco Cell Culture, Carlsbad, CA, USA), 1% penicillin-streptomycin (HyClone, Logan, UT, USA).

### 2.3. Transfection

The NCOA1-silenced (si-NCOA1) plasmid was constructed by Genechem (Shanghai, China) and the si-NCOA1 Ishikawa and RL95-2 cells were obtained through transfection. An empty vector that did not target any gene was used as a negative control (NC). The transfections were performed using Lipofectamine 3000 (Invitrogen, Thermo Fisher Scientific, Waltham, MA, USA) according to the manufacturer’s instructions. At 48 h post-transfection, we lysed the cells to extract the RNA and performed RT-qPCR. 

### 2.4. Immunohistochemistry (IHC)

Paraffin sections (5 μm) of normal endometrial (n = 6) and UEC tissues (n = 6) from the patients were dehydrated in graded ethanol and incubated with 3% hydrogen peroxide (H_2_O_2_) and 5% bovine serum albumin to block endogenous peroxidase activity. The samples were subsequently incubated with rabbit antihuman ALAS1 (1:200; cat. no. DF2900; Affinity, Cincinnati, OH, USA), antihuman SLC25A38 (1:200; cat. no. PA5-42472; Invitrogen), antihuman heme oxygenase 1 (1:100; cat. no. ab52947; Abcam, Cambridge, UK), or rabbit immunoglobulin G (IgG) isotype control (1:100; cat. no. ab125938; Abcam, negative control) overnight at 4 °C in a humid chamber. After washing three times with PBS, the sections were overlaid with peroxidase-conjugated goat antirabbit IgG, the reaction was developed using 3, 3-diaminobenzidine (DAB), and the sections were counterstained with hematoxylin. The immunohistochemical staining was quantified with integrated optical density values generated using Image-ProPlus 6.0 (Media Cybernetics, Rockville, MD, USA).

### 2.5. The Treatment of Estrogen, Estrogen Receptor (ER) Antagonist, Succinate, and Heme

The Ishikawa and RL95-2 cells were treated with estrogen (10^−7^ M, HY-B070, MedChem Express, NJ, USA), succinate (5 mM, Sigma-Aldrich; Merck KGaA, SL, MO, USA), ERα antagonist (MPP, 10^−6^ M, HY-103454, MedChem Express, Monmouth Junction, NJ, USA), ERβ antagonist (PHTPP, 10^−6^ M, HY-103456, MedChem Express, Monmouth Junction, NJ, USA), total ER antagonist Fulvestrant (ICI182780, 10^−6^ M, HY-13636, MedChem Express, Monmouth Junction, NJ, USA), and heme (0–100 μM, HY-19424, MedChem Express, Monmouth Junction, NJ, USA).

Both cell lines were seeded in 12-well plates at a density of 2.5 × 10^5^ cells/mL and cultured in DMEM/F12 medium (HyClone; GE Healthcare Life Sciences) supplemented with 10% FBS-charcoal-stripped (Gibco Cell Culture, Carlsbad, CA, USA) and 1% penicillin–streptomycin (HyClone, UT, USA). The relevant cells were cultured under 5% CO_2_ in a 37 °C humidified incubator (Heal Force HF-90, Shanghai, China) for 48 h.

### 2.6. Measurement of the Heme Concentration 

The heme concentrations were measured using a heme assay kit (cat. No. MAK316; Sigma-Aldrich; Merck KgaA). A total of 250 μL of distilled water was added to a blank well while the standard well contained 50 μL of heme calibrator and 200 μL of distilled water. A total of 50 μL of cell sample supernatant was mixed with 200 μL of reagent. The absorbance of the well contents was measured at 400 nm after incubation at room temperature for 5 min. All the samples and standards were performed in duplicate. The total heme concentration of a sample was calculated with the following formula: (OD_sample_ – OD_blank_)/(OD_calibrator_ – OD_blank_) × 62.5 (μmol/L).

### 2.7. RNA Extraction and Reverse Transcription Quantitative Real-Time Polymerase Chain Reaction (RT-qPCR)

The total RNA was extracted using TRIzol^®^ reagent (Invitrogen; Thermo Fisher Scientific, Inc., Waltham, MA, USA). RNA extraction was carried out on UEC tissues, paired adjacent healthy tissues, and two cell lines. The RNA was reverse-transcribed into cDNA using a Prime Script RT reagent kit (Takara Biotechnology Co., Ltd., Dalian, China). The qPCR was performed using an SYBR Premix Ex Taq kit (Takara Biotechnology Co., Ltd.) and analyzed using an ABI Prism 7900 Fast Sequence Detection system (Thermo Fisher Scientific, Inc.). The primer sequences of these genes are provided in Table 2. The fold change in the transcriptional expression of the above genes was calculated using the 2^−ΔΔCt^ method, and each sample was analyzed in three replicate wells. The relative mRNA expression levels were normalized to β-actin (ACTB).

### 2.8. Integration Analysis of the Protein–Protein Interaction (PPI) Network

The STRING database (http://string-db.org, accessed on 17 September 2022) was used for protein–protein interaction network prediction.

### 2.9. Cell Viability and Invasion Assays

After treatment with 0–100 μM of heme (HY-19424, MedChem Express, Monmouth Junction, NJ, USA) for 48 h, the viability and invasive ability of the Ishikawa and RL95-2 cells were analyzed via a Cell Counting Kit-8 (CCK-8) assay (Dojindo Molecular Technologies, Inc., Kumamoto, Japan) and a Matrigel (BD Biosciences, San Jose, CA, USA) invasion assay, respectively. We diluted the Matrigel to 1:30 and added 100 μL to each chamber. Then, we put the plate into a 37 °C incubator for 2 h, removed the excess liquid, and added 500 μL of serum-free culture into the lower chamber while adding the cells treated with heme for 48 h into the upper chamber. After another 48 h, we removed the excess liquid and wiped off the unpenetrated cells. We then fixed the cells with formaldehyde and stained them with crystal violet for 30 min, respectively. Finally, we observed the cells under a microscope and chose 4 fields of view for counting. The NC and si-NCOA1 cells were treated with or without 50 μM of heme for 48 h, and then the cell viability and invasiveness were analyzed again.

### 2.10. Statistical Analysis

The data are presented as means ± standard errors of the mean (means ± SEMs). Normality of the data was tested with a Shapiro–Wilk test, and the differences were statistically analyzed using Student’s *t*-tests or one-way ANOVA. Kruskal–Wallis tests were performed to assess the differences in the variables with skewed distribution between two groups or among the groups, respectively. Statistical analysis was performed using SPSS version 26.0, and a *p* value of < 0.05 was considered to indicate a statistically significant difference. All experiments were repeated at least three times.

## 3. Results

### 3.1. The Expression of ALAS1 and SLC25A38 in Primary Tumors Is Significantly Higher than Those in Normal Tissues

**Figure 1:**
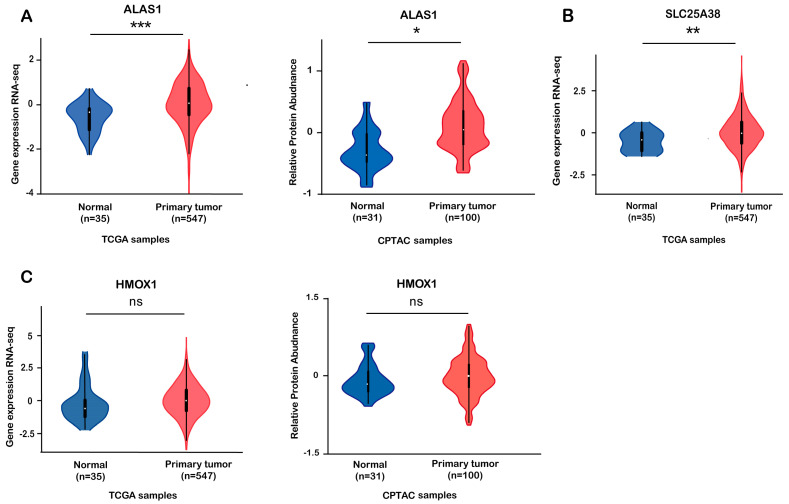
TCGA and CPTAC public databases showing that active heme synthesis can be observed in endometrial cancer tissues. (**A**–**C**) The expression of ALAS1, SLC25A38, and HMOX1 in primary tumor samples and normal control endometrium tissues. Statistical significance (*t*-tests or Kruskal-Wallis tests with Dunn’s multiple comparison tests): *: *p* < 0.05; **: *p* < 0.01; ***: *p* < 0.001; ns: no significant difference.

To investigate the expression of heme metabolism-related molecules in UEC, we used The Cancer Genome Atlas (TCGA, https://cancergenome.nih.gov/, accessed on 20 August 2022) and Clinical Proteomic Tumor Analysis Consortium (CPTAC, https://gdc.cancer.gov/, accessed on 20 August 2022) public databases for our analysis. As shown, the expression of the key enzyme ALAS1 and the glycine mitochondrial transporter SLC25A38 in the primary tumors was significantly higher than that of the normal tissues (Figure 1A,B), though there was no difference in the expression of HMOX1, which mediates heme degradation (Figure 1C).

**Figure 2:**
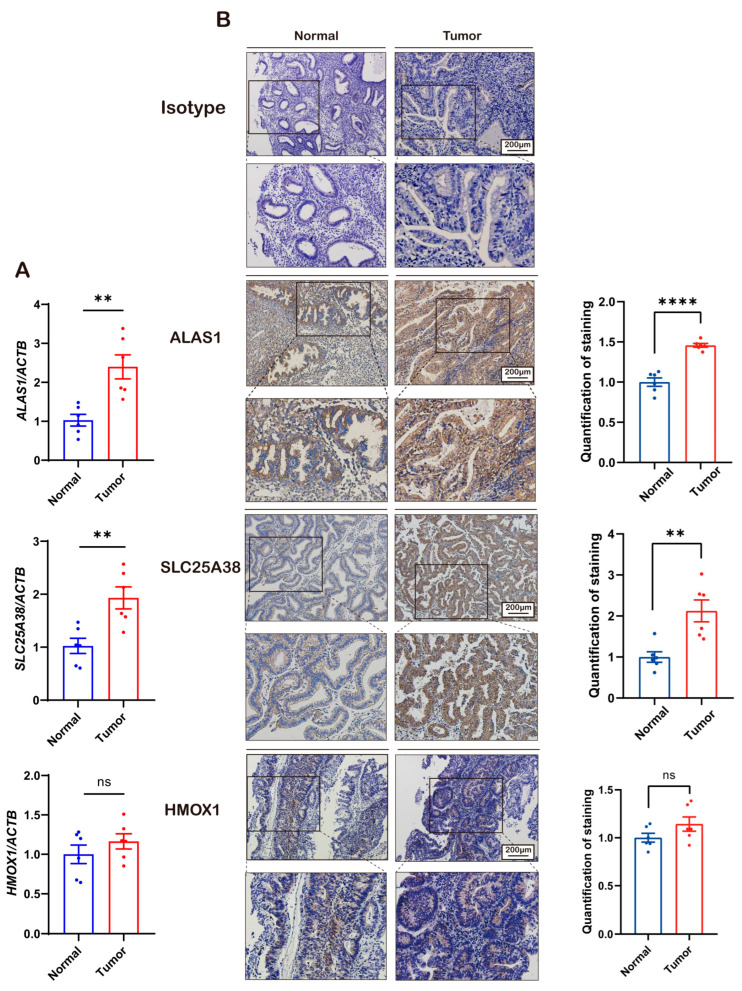
The higher expression of ALAS1 and SLC25A38 observed in endometrial cancer tissues. (**A**) Relative mRNA expression of ALAS1, SLC25A38, and HMOX1 in human UEC tissues and adjacent matching healthy endometrium tissues. (**B**) Left: IHC staining of isotype control, ALAS1, SLC25A38, and HMOX1 expression in the two types of tissues. Right: quantification of the IHC results for the six pairs of samples. The immunohistochemical staining was quantified with integrated optical density values generated using Image-ProPlus 6.0 (Media Cybernetics, Rockville, MD, USA). The data shown are means ± SEMs or medians with 95% confidence intervals. Statistical significance (*t*-tests or Kruskal–Wallis tests with Dunn’s multiple comparison tests): **: *p* < 0.01; ****: *p* < 0.0001; ns: no significant difference.

Then, we collected six pairs of UEC samples and their adjacent healthy tissues, and RT-qPCR and immunohistochemistry were used to further verify the results of the public databases (Figure 2A,B). These results suggested that heme synthesis in UEC is extremely active, and it is apt to form a high-heme microenvironment.

### 3.2. Estrogen and Succinate Increase the Expression of ALAS1 and SLC25A38 in UECC

**Figure 3:**
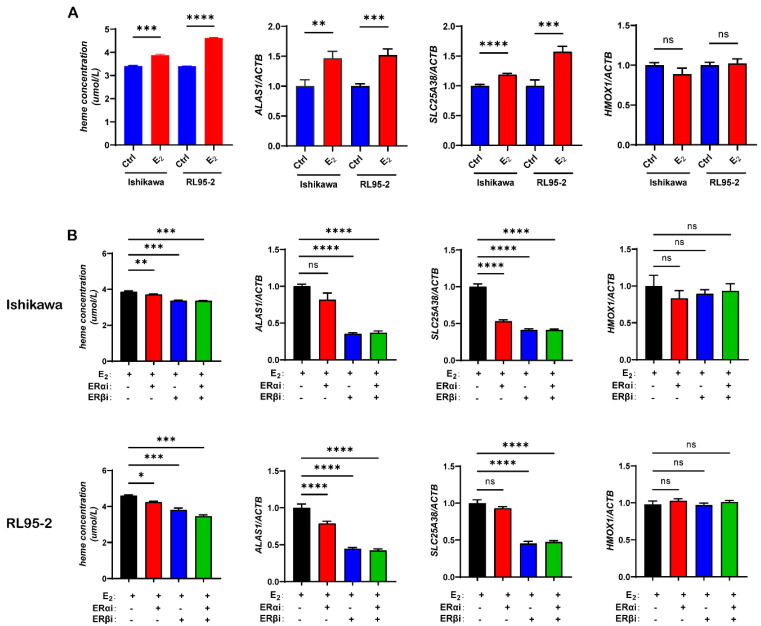
The heme concentrations and expression levels of metabolism-related molecules in Ishikawa and RL95-2 cells are significantly increased with the administration of estrogen, though ERβi, specifically, can block this effect. (**A**) Heme concentrations and relative mRNA expression levels of ALAS1, SLC25A38, and HMOX1 in the Ishikawa and RL95-2 cells treated with or without 10^−7^ M estrogen (E_2_) for 48 h. (**B**) Heme concentrations and relative mRNA expression levels of ALAS1, SLC25A38, and HMOX1 in the Ishikawa and RL95-2 cells treated with 10^−7^ M E_2_, 10^−7^ M E_2_, and 10^−6^ M ERα antagonist MPP (ERαi) and 10^−6^ M ERβ antagonist PHTPP (ERβi) or 10^−6^ M total ER antagonist ICI182780 (ERαi plus ERβi) for 48 h. The data shown are means ± SEMs analyzed using *t*-tests or one-way ANOVA tests. *: *p* < 0.05; **: *p* < 0.01; ***: *p* < 0.001; ****: *p* < 0.0001; ns: no significant difference.

To explore the effect of estrogen on heme synthesis in UECC, we treated the Ishikawa and RL95-2 cells with or without 10^−7^ M estrogen for 48 h, and then we detected the heme concentrations and the expression levels of ALAS1, SLC25A38, and HMOX1. As shown, we found that estrogen treatment significantly upregulated the expression of ALAS1 and SLC25A38, while there was no difference in the expression of HMOX1. As a result, we confirmed estrogen regulation of heme expression (Figure 3A).

After adding 10^−6^ M ERαi, ERβi, or total ER antagonist (ERαi + ERβi), the concentrations of heme in the cells were significantly reduced, and the blocking effect of ERβi appeared to be more significant and specific (Figure 3B).

**Figure 4:**
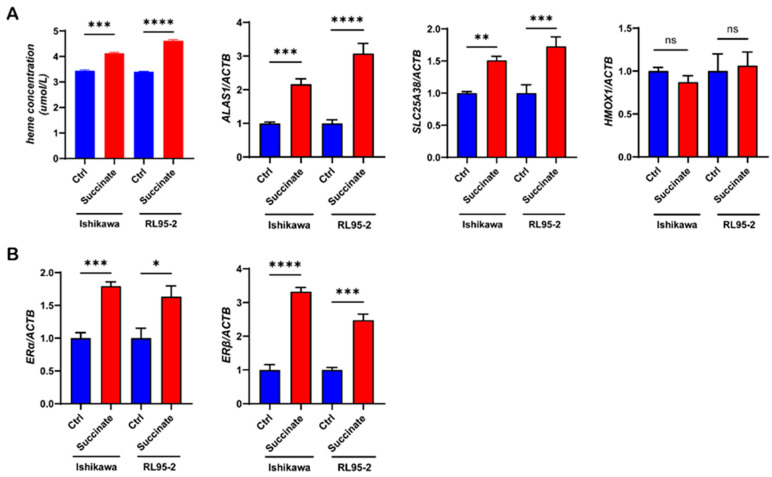
The heme concentrations and expression levels of metabolism-related molecules in Ishikawa and RL95-2 cells are significantly increased with the administration of succinate, and succinate can significantly upregulate the level of ER. (**A**) Heme concentrations and relative mRNA expression levels of ALAS1, SLC25A38, and HMOX1 in the Ishikawa and RL95-2 cells treated with or without 5 mM succinate for 48 h. (**B**) Relative mRNA expression levels of ERα and ERβ in the Ishikawa and RL95-2 cells treated with or without 5 mM succinate for 48 h. The data shown are means ± SEMs analyzed using *t*-tests. *: *p* < 0.05; **: *p* < 0.01; ***: *p* < 0.001; ****: *p* < 0.0001; ns: no significant difference.

Subsequently, we treated the Ishikawa and RL95-2 cells with or without 5 mM succinate for 48 h, and measured the expression levels of ALAS1, SLC25A38, and HMOX1. Similarly, we found that succinate significantly upregulated the expression of ALAS1 and SLC25A38. As expected, heme levels were increased with the administration of succinate (Figure 4A). Further, ER expression was also significantly upregulated following succinate treatment (Figure 4B).

Together, these data indicated that the administration of estrogen or succinate significantly upregulated the expression of heme metabolism-related molecules and increased the concentrations of heme in UECC.

### 3.3. Estrogen Increases the Expression of ALAS1 and the Synthesis of Heme by the ERβ/NCOA1 Axis

**Figure 5:**
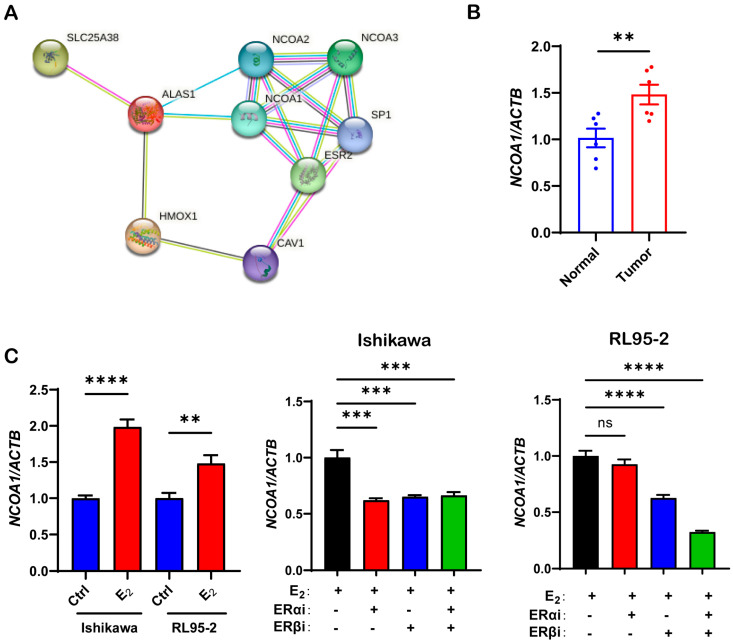
The protein-protein interaction network analysis showed that NCOA1 may be a key molecule in the regulation of estrogen-heme metabolism. (**A**) Differentially expressed gene-encoded protein-protein interaction network based on the STRING database. (**B**) Relative mRNA expression levels of NCOA1 in human UEC tissues and adjacent matching healthy endometrium tissues. (**C**) Relative mRNA expression levels of NCOA1 in the Ishikawa and RL95-2 cells treated with 10^−7^ M E_2_, 10^−7^ M E_2_, and 10^−6^ M ERαi and ERβi or nonselective ERi for 48 h. The data shown are means ± SEMs analyzed using *t*-tests or one-way ANOVA tests. **: *p* < 0.01; ***: *p* < 0.001; ****: *p* < 0.0001; ns: no significant difference.

To explore the possible molecular mechanisms in estrogen-regulated heme synthesis, a protein–protein interaction network analysis was performed. As shown in Figure 5A, nuclear receptor coactivator 1 (NCOA1) may be a key molecule between ER and heme synthesis-related molecules (ALAS1 and SLC25A38) (Figure 5A). It has been reported that NCOA1 overexpression can be observed in a variety of pathological conditions, such as diabetes, obesity, and tumors [22,23].

Subsequently, we used RT-qPCR to verify the that the expression of NCOA1 in UEC was significantly higher than that in normal tissues (Figure 5B). We treated Ishikawa and RL95-2 cells with 10^−7^ M estrogen or 10^−6^ M ERi for 48 h and quantified the relative mRNA expression of NCOA1. We found that estrogen treatment increased the expression of NCOA1 whereas 10^−6^ M ERαi, ERβi, or nonselective ERi significantly reduced the expression of NCOA1 (Figure 5C), suggesting that the NCOA1 gene is regulated by the estrogen pathway. Therefore, we speculated that there may be an interactive regulatory relationship between estrogen, succinate, and NCOA1 in UEC.

**Figure 6:**
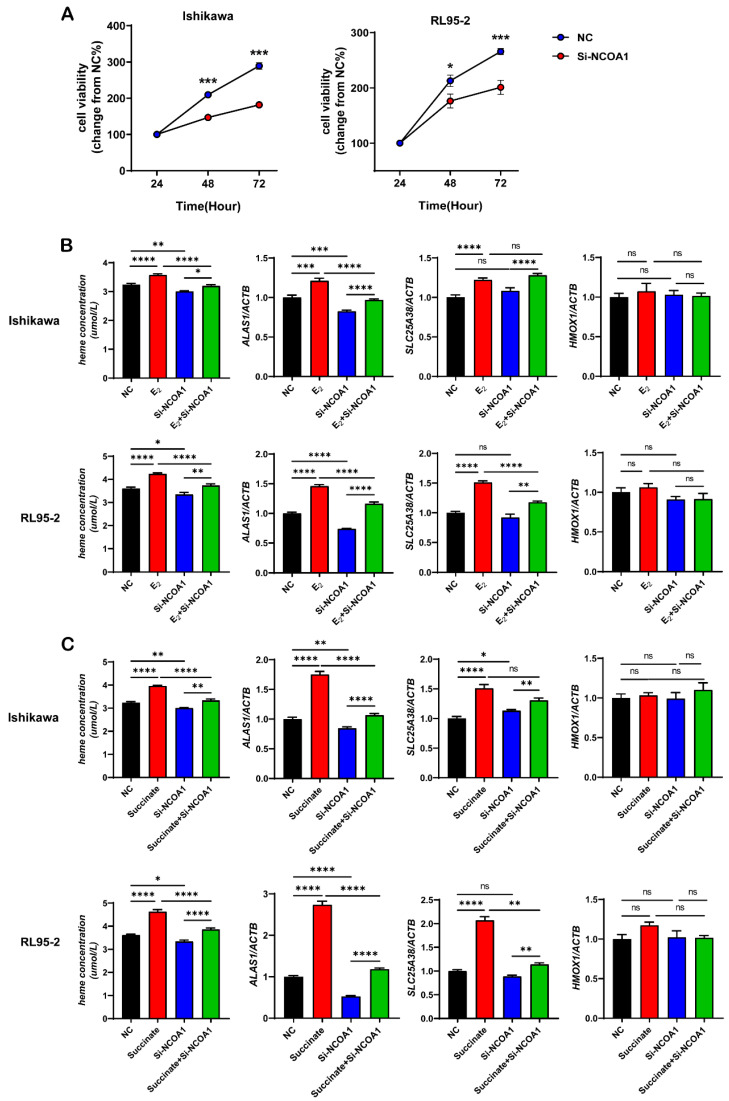
Silencing NCOA1 decreases the viability of Ishikawa and RL95-2 cells and downregulates the expression of ALAS1. (**A**) Cell viability of the NC or si-NCOA1 Ishikawa and RL95-2 cells after 24, 48, or 72 h were detected using a Cell Counting Kit-8 assay. (**B**,**C**) Heme concentrations and relative mRNA expression levels of ALAS1, SLC25A38, and HMOX1 in the NC or si-NCOA1 Ishikawa and RL95-2 cells treated with 10^−7^ M E_2_ (**B**) or 5 mM succinate (**C**) for 48 h. The data shown are means ± SEMs analyzed using *t*-tests or one-way ANOVA tests. *: *p* < 0.05; **: *p* < 0.01; ***: *p* < 0.001; ****: *p* < 0.0001; ns: no significant difference.

To clarify whether NCOA1 has a regulatory effect on the proliferation and invasion of UECC, we constructed a negative control (NC) and NCOA1-silenced (si-NCOA1) plasmids, and transfected the Ishikawa and RL95-2 cells with them, respectively. We detected that the cell viability at 24, 48, and 72 h was significantly reduced following the silencing of NCOA1 (Figure 6A).

Next, 10^−7^ M estrogen, 10^−6^ M Eri, or 5 mM of succinate was used to treat the NC or the si-NCOA1 Ishikawa and RL95-2 cells for 48 h. We detected the heme concentrations and the expression levels of the heme metabolism-related molecules. As shown, we found that compared with the NC, the heme concentration and the expression of ALAS1 were significantly decreased in the si-NCOA1 cells while there were no differences in the expression levels of SLC25A38 and HMOX1. Moreover, although estrogen and succinate could increase heme synthesis in UECC, silencing NCOA1 partially or completely eliminated this effect (Figure 6B,C).

### 3.4. Exposure to Heme Increases the Viability and Invasiveness of UECC

**Figure 7:**
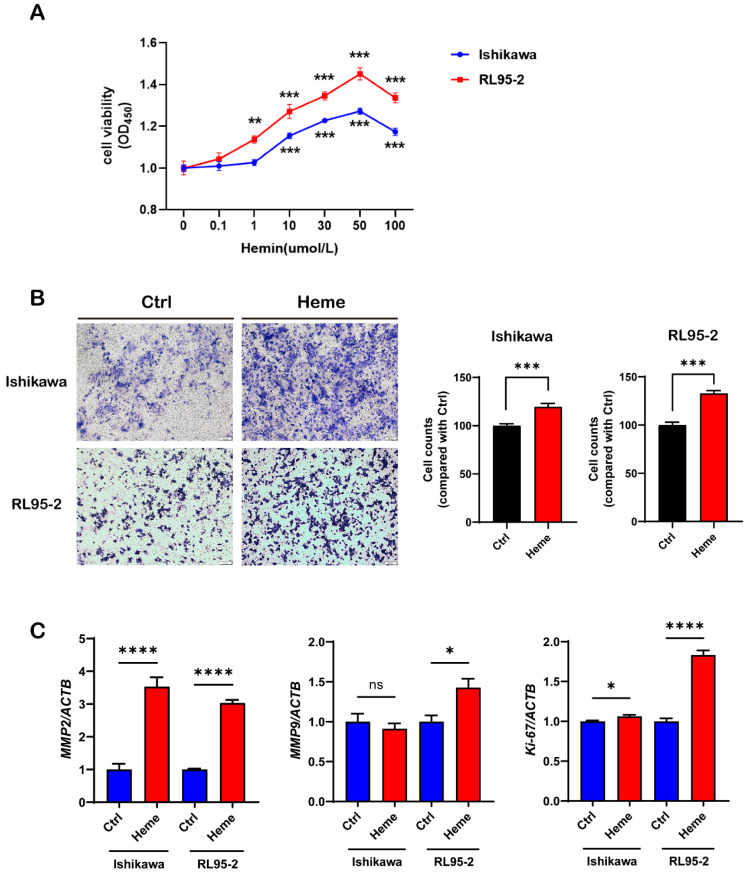
The cell viability, invasiveness, and proliferation of Ishikawa and RL95-2 cells are significantly increased with the administration of heme. (**A**) The cell viability of the Ishikawa and RL95-2 cells after treatment with different concentrations of heme (0, 0. 1, 1, 10, 30, 50, and 100 μM) was analyzed using a Cell Counting Kit-8 assay. (**B**) The cell invasiveness of Ishikawa and RL95-2 cells treated with or without heme (50 μM) for 48 h was analyzed using a Matrigel invasion assay. (**C**) The relative mRNA expression levels of MMP2, MMP9, and Ki-67 in Ishikawa and RL95-2 cells treated with or without 50 μM heme for 48 h. The data shown are the means ± SEMs analyzed using *t*-tests or one-way ANOVA tests. *: *p* < 0.05; **: *p* < 0.01; ***: *p* < 0.001; ****: *p* < 0.0001; ns: no significant difference.

To further analyze the mechanism of heme in accelerating the progression of UEC, we treated Ishikawa and RL95-2 cells with different concentrations of heme (0, 0.1, 1, 10, 30, 50, and100 μM) for 48 h and detected cell viability using a CCK8 assay. We found that cell viability was significantly increased with the administration of heme of up to 50 μM (Figure 7A).

Through destroying the basement membrane of cells, matrix metalloproteinases (MMPs) allow tumor cells to enter the blood and lymphatic vessels, leading to the invasion and metastasis of tumors [24,25]. Ki-67 is present in the G1, S, and G2 phases of the cell cycle rather than in the G0 phase, where cells stop dividing [26]. Therefore, Ki-67 has been widely used as a proliferative marker to assess the proliferative activity of cells. We treated Ishikawa and RL95-2 cells with 50 μM of heme for 48 h. Invasiveness was analyzed using a Matrigel invasion assay, and the expression levels of MMP2, MMP9, and Ki-67 were analyzed using RT-qPCR. We determined that the invasiveness of UECC was increased with the administration of heme, and the expression levels of MMP2, MMP9, and Ki-67 were also upregulated (Figure 7B,C). In summary, exposure to heme promoted the proliferation and invasiveness of UECC.

### 3.5. Silencing the NCOA1 Gene Weakens the Effect of Heme on Promoting Cell Viability and Invasiveness in UECC

**Figure 8:**
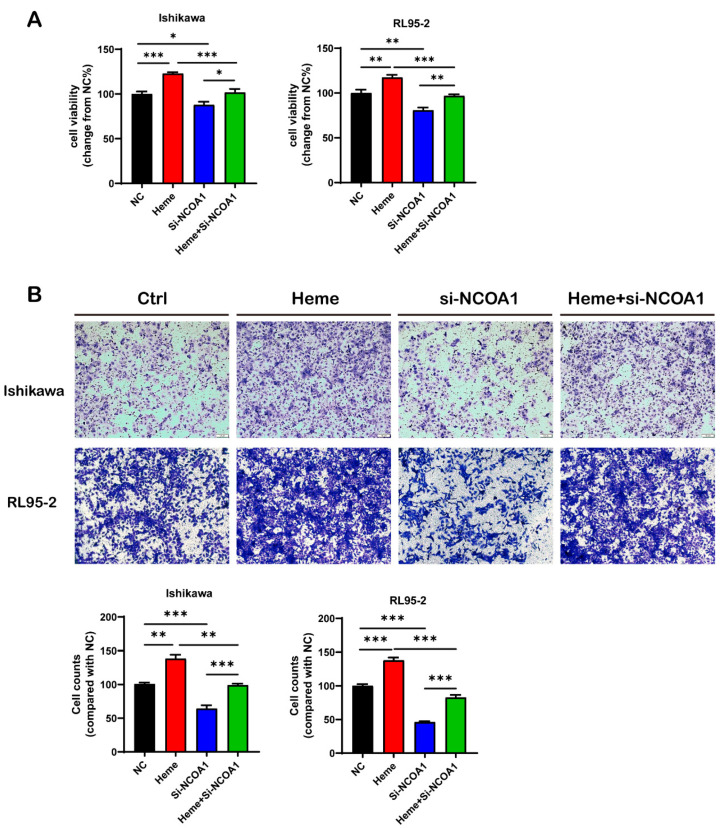
Silencing NCOA1 decreases the viability and invasiveness of Ishikawa and RL95-2 cells treated with heme. (**A**) The cell viability of the NC or si-NCOA1 cells after treatment with or without 50 μM heme was analyzed using a CCK8 assay. (**B**) The cell invasiveness of the NC or si-NCOA1 cells treated with or without 50 μM heme for 48 h was analyzed using a Matrigel invasion assay. The data shown are means ± SEMs analyzed using one-way ANOVA tests. *: *p* < 0.05; **: *p* < 0.01; ***: *p* < 0.001.

We treated NC or si-NCOA1 Ishikawa and RL95-2 cells with or without 50 μM heme for 48 h, and cell viability and invasiveness were detected using CCK8 and Matrigel invasion assays. We found that, compared with the NC, silencing NCOA1 significantly reduced the cell viability and invasiveness of UECC. In addition, although heme could promote the cell viability and invasiveness of UECC, this effect could be partially or completely eliminated in the si-NCOA1 cells (Figure 8A,B).

## 4. Discussion

Heme is a complex of iron and protoporphyrin IX. It plays a crucial role in biosynthesis in the form of covalent or noncovalent binding [18,27]. Heme b participates in the synthesis of hemoglobin and myoglobin in a noncovalent binding form, which is of great importance for the reversible binding and transportation of oxygen. Heme c forms covalent binding with cytochrome c and is involved in electron transfer in the mitochondrial respiratory chain [28]. Heme is also part of the prosthetic group for many key enzymes in maintaining cellular homeostasis, such as inducible nitric oxide synthase (iNOS), cytochrome P450, and soluble guanylate cyclase (sGC) [29]. In addition, there are small amounts of free heme in cells, which are known as unstable heme pools, and which also have a regulatory effect on cellular metabolism. For example, they act as important signaling molecules that regulate circadian rhythms [30]. Due to the important role heme plays in cell metabolism, maintaining heme homeostasis is of great physiological significance. The levels of free heme in cells depend on the expression of heme metabolism-related molecules [15].

ALAS1 is a key enzyme in heme biosynthesis [16]. As a housekeeping gene, ALAS1 is widely present in almost all tissues. Impaired heme synthesis can lead to the accumulation of the toxic heme intermediate δ-amino-γ-ketovaleric acid (ALA), which can eventually lead to porphyria [17]. Solute carrier family 25 A38 (SLC25A38), which is also known as a glycine mitochondrial transporter, can transport exogenous glycine into mitochondria for biosynthesis [17]. In the TCA cycle, α-ketoglutaric acid produces succinyl CoA under the action of dehydrogenase while glycine is transported into mitochondria through SLC25A38. Succinyl CoA binds to glycine via ALAS1, and after a series of complex reactions, finally, heme is generated [18,19]. HMOX1 is a key enzyme in mediating heme degradation, and it exists in almost all mammalian tissues. Under normal physiological circumstances, free heme can be degraded by HMOX1 into iron, carbon monoxide, and biliverdin [19,20]. 

Through analyzing the TCGA and CPTAC public databases, we found that the expression levels of ALAS1 and SLC25A38 in primary tumors are significantly higher than those in normal tissues, and there were no significant differences in the expression levels of HMOX1 in the two types of tissues. The results of the clinical samples were also consistent with the public databases.

Estrogen can regulate the activity of various tumor-associated enzymes such as liver ribonucleases and glutaminase (GLS). Estrogen also plays a crucial role in the progression of type I UEC [6,31]. Studies have shown that by upregulating the expression of the proto-oncogene c-MYC, estrogen can significantly increase the level of GLS, resulting in increased cell viability and decreased autophagy in estrogen-sensitive UEC [8]. In our study, we found that heme concentrations and the expression levels of metabolism-related molecules in UECC are significantly increased with the administration of succinate or estrogen. We also observed that succinate can upregulate the expression of two ERs, especially that of ERβ. Not surprisingly, adding ERi reduced the concentrations of heme in the cells while the blocking effect of ERβi was more significant and specific.

The expression of genes is precisely regulated by transcriptional complexes that consist of transcription factors and transcriptional regulators [32]. Rather than regulating the target gene directly, transcriptional regulators must rely on a corresponding transcription factor to play a role [33,34]. NCOA1, also known as steroid receptor coactivator 1, can stimulate transcription in a hormone-dependent manner by binding to nuclear receptors [23,35]. We used a protein–protein interaction network analysis and found that NCOA1 may be a key molecule in the process in which estrogen regulates succinate–heme metabolism. It has been reported that NCOA1 overexpression has been observed in a variety of pathological conditions, such as diabetes, obesity, and carcinogenesis. The increased expression of NCOA1 in bladder cancer patients is positively correlated with the tumor clinical stage, the pathological grade, and lymph node metastasis. Poorer outcomes may be due to NCOA1 promotion of cell proliferation through upregulating the level of androgen receptors [36,37].

We found that silencing NCOA1 decreased cell viability and downregulated the expression of ALAS1, suggesting that estrogen may promote heme biosynthesis by activating the expression of NOCA1 and increasing ALAS1 synthesis. In addition, although estrogen and succinate can increase the levels of heme in Ishikawa and RL95-2 cells, silencing NCOA1 can partially or even completely eliminate this effect. This further confirmed that NCOA1 may be an important regulatory molecule in estrogen–succinate–heme metabolism. 

The occurrence of breast cancer is also closely related to estrogen. Studies have verified that estrogen can upregulate the expression of NCOA1 through ER. The combination of NCOA1 and the transcription factor HOXC11 can activate the transcription of downstream genes, including integrin α5, ADAM22, and Myc, and can thus promote the metastasis and progression of breast cancer [38]. The mitogen-activated protein kinase (MAPK) signal pathway is a key kinase that participates in the regulation of cell proliferation and apoptosis. It has been confirmed that the activation of the MAPK pathway can lead to the translocation of nuclear transcription factor κB (NF-κB) from the cytoplasm to the nucleus and upregulate the expression of NF-κB downstream molecules, such as interleukin-1β (IL1β), IL6, and tumor necrosis factor-α [39]. Peng et al. found that the levels of NCOA1 in patients with myocardial dysfunction were significantly increased. Further studies showed that NCOA1 can form a transcriptional complex with NF-κB, and thus activates the expression of proinflammatory-related cytokines, such as in the myocardium [22]. However, the exact regulatory mechanisms of estrogen on NCOA1 and of the ERβ/NCOA1 axis on ALAS1 in UEC need to be explored further.

A growing volume of studies have suggested that heme metabolism plays a key role in the pathogenesis of various diseases. Some scholars have already proposed that heme-targeted drugs may be used as potential angiogenesis inhibitors and are expected to be new treatment strategies for many diseases in the future [40]. Heme-regulated inhibitor (HRI), also known as eIF2AK1, is the member of eukaryotic translation initiation factor 2 alpha kinase (eIF2AK) family. It has been reported that heme could affect protein biosynthesis via phosphorylating α subunit of eIF2 on serine 51 residue [41]. HRI has been proven to increase fetal hemoglobin production via diminishing the expression of BCL11A, and this may be a potential therapeutic target for sickle cell disease [42]. Moreover, mifepristone activates cytotoxic effects through the eIF2AK1-mediated signal transduction pathway, promoting the drug synergies in ovarian cancer therapy [43]. In our study, we treated UECC with different concentrations of heme and found that heme increased the cell viability and invasiveness of UECC while, as expected, silencing NCOA1 could partially or even completely eliminate this effect of heme. In addition, our previous study confirmed that excess heme impairs the phagocytosis of peritoneal macrophages, promoting the progression of endometriosis [44]. We speculated that the large amounts of heme present in UEC may also have similar functions. However, follow-up in vivo experiments still need to be conducted. Moreover, the underlying molecular mechanisms of how heme regulates cell biological behavior also deserve to be further studied.

## 5. Conclusions

**Figure 9:**
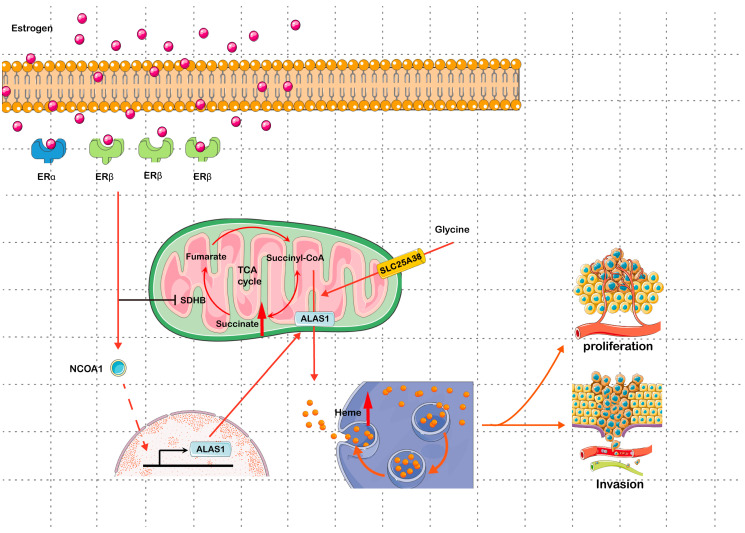
The mechanisms of estrogen–succinate–heme metabolism in uterine endometrial cancer. (1) Estrogen can inhibit SDHB expression through the PPARγ-SP1-UBC pathway, promoting the accumulation of succinate in UEC. The abnormal accumulation of succinate promotes heme synthesis and upregulates ER levels, especially those of ERβ. (2) Estrogen activates the expression of NCOA1 mainly through ERβ, increasing the synthesis of ALAS1. (3) Glycine can be transported into the mitochondria under the mediation of SLC25A38 and eventually participate in heme biosynthesis. Estrogen and succinate synergistically increase the accumulation of free heme, which promotes the proliferation and invasiveness of cells, accelerating the progression of UEC. SDHB: succinate dehydrogenase B; PPARγ: peroxisome proliferator-activated receptor γ; SP1: specificity protein 1; UBC: ubiquitin C; UEC: uterine endometrial cancer; ER: estrogen receptor; NCOA1: nuclear receptor coactivator 1; ALAS1: aminolevulinate delta synthase 1; SLC25A38: solute carrier family 25 A38.

In this study, as shown in Figure 9, we found that there are high levels of heme in UECC. By activating the expression of NCOA1 through ERs, estrogen increases the expression of ALAS1 and promotes heme synthesis in UECC. Meanwhile, the low levels of SDHB in UEC lead to the abnormal accumulation of succinate, which also promotes heme synthesis and upregulates ER levels. Estrogen and succinate synergistically promote the accumulation of free heme, increasing the proliferative and invasive potential of UECC. In summary, our study tried to clarify the characteristics of estrogen–succinate–heme metabolism in UEC progression. We believe this study will provide a theoretical basis and potential target for the anticancer treatment of UEC in the future.

## Figures and Tables

**Table 1:** The baseline characteristics of the UEC patients.

Case	Age	Menopausal	Menstrual Phase	Type	Pathological Type	Grade	Stage	TNM
1	55	Yes	/	I	Endometrioid carcinoma	G1	IA	T1aNsn0M0
2	52	Yes	/	I	Endometrioid carcinoma	G1	IA	T1aNsn0M0
3	55	Yes	/	I	Endometrioid carcinoma	G1	IB	T1bNsn0M0
4	37	No	Secretory phase	I	Endometrioid carcinoma	G1	IIIC2	T1aN2aM0
5	33	No	Secretory phase	I	Endometrioid carcinoma	G1	IA	T1aNsn0M0
6	52	Yes	/	I	Endometrioid carcinoma	G2	IIIA	T3aN0M0

**Table 2:** The sequences of the primers used for RT-qPCR.

Gene	Sequence
ATCB	Forward: GCCGACAGGATGCAGAAGGAGATCA
	Reverse: AAGCATTTGCGGTGGACGATGGA
ALAS1	Forward: TCAACCCTCTTCACCCTGGCTAAG
	Reverse: TACTTTGGCACTCGGCTGTTTCG
SLC25A38	Forward: ATGATTCAGAACTCACGTCCGT
	Reverse: CAGGCGTGTTTTAAGGAGATCC
HMOX1	Forward: TGCCAGTGCCACCAAGTTCAAG
	Reverse: TGTTGAGCAGGAACGCAGTCTTG
ERα	Forward: GAAAGGTGGGATACGAAAAGACC
	Reverse: GCTGTTCTTCTTAGAGCGTTTGA
ERβ	Forward: TTCAAAGAGGGATGCTCACTTC
	Reverse: CCTTCACACGACCAGACTCC
NCOA1	Forward: AATGAATACGAGCGTCTACAGC
	Reverse: TTTCGTCGTGTTGCCTCTTGA
MMP2	Forward: TACAGGATCATTGGCTACACACC
	Reverse: GGTCACATCGCTCCAGACT
MMP9	Forward: AGACCTGGGCAGATTCCAAAC
	Reverse: CGGCAAGTCTTCCGAGTAG
Ki-67	Forward: ACGCCTGGTTACTATCAAAAGG
	Reverse: CAGACCCATTTACTTGTGTTGGA

## Data Availability

Not applicable.

## References

[1] Dashti S.G., Simpson J.A., Viallon V., Karahalios A., Moreno-Betancur M., Brasky T., Pan K., Rohan T.E., Shadyab A.H., Thomson C.A. (2022). Adiposity and breast, endometrial, and colorectal cancer risk in postmenopausal women: Quantification of the mediating effects of leptin, C-reactive protein, fasting insulin, and estradiol. Cancer Med..

[2] Braun M.M., Overbeek-Wager E.A., Grumbo R.J. (2016). Diagnosis and Management of Endometrial Cancer. Am. Fam. Physician.

[3] Onstad M.A., Schmandt R.E., Lu K.H. (2016). Addressing the Role of Obesity in Endometrial Cancer Risk, Prevention, and Treatment. J. Clin. Oncol..

[4] Bidzinski M., Danska-Bidzinska A., Rychlik A., Kypryjanczyk J., Pyzlak M., Piatek S. (2022). Molecular classification of endometrial carcinoma, is it the new era of precision medicine?. Ginekol. Pol..

[5] Liang Y., Jiao H., Qu L., Liu H. (2021). Association Between Hormone Replacement Therapy and Development of Endometrial Cancer: Results From a Prospective US Cohort Study. Front. Med..

[6] Amant F., Moerman P., Neven P., Timmerman D., Van Limbergen E., Vergote I. (2005). Endometrial cancer. Lancet.

[7] Passarello K., Kurian S., Villanueva V. (2019). Endometrial Cancer: An Overview of Pathophysiology, Management, and Care. Semin. Oncol. Nurs..

[8] Zhou W.J., Zhang J., Yang H.L., Wu K., Xie F., Wu J.N., Wang Y., Yao L., Zhuang Y., Xiang J.D. (2019). Estrogen inhibits autophagy and promotes growth of endometrial cancer by promoting glutamine metabolism. Cell Commun. Signal..

[9] Li J., Tuo Z., Zong Y., Liu J. (2021). Succinate dehydrogenase 5 regulates lung cancer metastasis by reprogramming glucose metabolism. J. Thorac. Dis..

[10] Bian X., Liu R., Meng Y., Xing D., Xu D., Lu Z. (2021). Lipid metabolism and cancer. J. Exp. Med..

[11] Martínez-Reyes I., Chandel N.S. (2020). Mitochondrial TCA cycle metabolites control physiology and disease. Nat. Commun..

[12] Eniafe J., Jiang S. (2021). The functional roles of TCA cycle metabolites in cancer. Oncogene.

[13] Imperiale A., Moussallieh F.M., Roche P., Battini S., Cicek A.E., Sebag F., Brunaud L., Barlier A., Elbayed K., Loundou A. (2015). Metabolome profiling by HRMAS NMR spectroscopy of pheochromocytomas and paragangliomas detects SDH deficiency: Clinical and pathophysiological implications. Neoplasia.

[14] Gu C., Yang H., Chang K., Zhang B., Xie F., Ye J., Chang R., Qiu X., Wang Y., Qu Y. (2020). Melatonin alleviates progression of uterine endometrial cancer by suppressing estrogen/ubiquitin C/SDHB-mediated succinate accumulation. Cancer Lett..

[15] Ryter S.W., Tyrrell R.M. (2000). The heme synthesis and degradation pathways: Role in oxidant sensitivity. Heme oxygenase has both pro- and antioxidant properties. Free Radic. Biol. Med..

[16] Yien Y.Y., Perfetto M. (2022). Regulation of Heme Synthesis by Mitochondrial Homeostasis Proteins. Front. Cell Dev. Biol..

[17] Peoc’h K., Nicolas G., Schmitt C., Mirmiran A., Daher R., Lefebvre T., Gouya L., Karim Z., Puy H. (2019). Regulation and tissue-specific expression of delta-aminolevulinic acid synthases in non-syndromic sideroblastic anemias and porphyrias. Mol. Genet. Metab..

[18] Kafina M.D., Paw B.H. (2017). Intracellular iron and heme trafficking and metabolism in developing erythroblasts. Metallomics.

[19] Creeden J.F., Gordon D.M., Stec D.E., Hinds T.D. (2021). Bilirubin as a metabolic hormone: The physiological relevance of low levels. Am. J. Physiol. Endocrinol. Metab..

[20] Ali F.F., Abdelzaher W.Y., Ibrahim R.A., Elroby Ali D.M. (2019). Amelioration of estrogen-induced endometrial hyperplasia in female rats by hemin via heme-oxygenase-1 expression, suppression of iNOS, p38 MAPK, and Ki67. Can. J. Physiol. Pharmacol..

[21] Ruan L.Y., Lai Z.Z., Shi J.W., Yang H.L., Ye J.F., Xie F., Qiu X.M., Zhu X.Y., Li M.Q. (2022). Excess Heme Promotes the Migration and Infiltration of Macrophages in Endometrial Hyperplasia Complicated with Abnormal Uterine Bleeding. Biomolecules.

[22] Peng Q., Hua Y., Xu H., Chen X., Xu H., Wang L., Zhao H. (2022). The NCOA1-CBP-NF-kappaB transcriptional complex induces inflammation response and triggers endotoxin-induced myocardial dysfunction. Exp. Cell Res..

[23] Wang L., Li W., Li K., Guo Y., Liu D., Yao Z., Lin X., Li S., Jiang Z., Liu Q. (2018). The oncogenic roles of nuclear receptor coactivator 1 in human esophageal carcinoma. Cancer Med..

[24] Muniz-Bongers L.R., McClain C.B., Saxena M., Bongers G., Merad M., Bhardwaj N. (2021). MMP2 and TLRs modulate immune responses in the tumor microenvironment. JCI Insight.

[25] Liu D., Kang H., Gao M., Jin L., Zhang F., Chen D., Li M., Xiao L. (2020). Exosome-transmitted circ_MMP2 promotes hepatocellular carcinoma metastasis by upregulating MMP2. Mol. Oncol..

[26] Menon S.S., Guruvayoorappan C., Sakthivel K.M., Rasmi R.R. (2019). Ki-67 protein as a tumour proliferation marker. Clin. Chim. Acta.

[27] Dutt S., Hamza I., Bartnikas T.B. (2022). Molecular Mechanisms of Iron and Heme Metabolism. Annu. Rev. Nutr..

[28] Immenschuh S., Vijayan V., Janciauskiene S., Gueler F. (2017). Heme as a Target for Therapeutic Interventions. Front. Pharmacol..

[29] Wijayanti N., Kietzmann T., Immenschuh S. (2005). Heme oxygenase-1 gene activation by the NAD(P)H oxidase inhibitor 4-(2-aminoethyl) benzenesulfonyl fluoride via a protein kinase B, p38-dependent signaling pathway in monocytes. J. Biol. Chem..

[30] Canesin G., Muralidharan A.M., Swanson K.D., Wegiel B. (2021). HO-1 and Heme: G-Quadruplex Interaction Choreograph DNA Damage Responses and Cancer Growth. Cells.

[31] Dunneram Y., Greenwood D.C., Cade J.E. (2019). Diet, menopause and the risk of ovarian, endometrial and breast cancer. Proc. Nutr. Soc..

[32] Mahajan V., Gujral P., Jain L., Ponnampalam A.P. (2022). Differential Expression of Steroid Hormone Receptors and Ten Eleven Translocation Proteins in Endometrial Cancer Cells. Front. Oncol..

[33] Chen X., Liu Z., Xu J. (2010). The cooperative function of nuclear receptor coactivator 1 (NCOA1) and NCOA3 in placental development and embryo survival. Mol. Endocrinol..

[34] Miki Y., Iwabuchi E., Takagi K., Suzuki T., Sasano H., Yaegashi N., Ito K. (2022). Co-expression of nuclear heterogeneous nuclear ribonucleic protein K and estrogen receptor alpha in endometrial cancer. Pathol. Res. Pract..

[35] Qin L., Xu Y., Xu Y., Ma G., Liao L., Wu Y., Li Y., Wang X., Wang X., Jiang J. (2015). NCOA1 promotes angiogenesis in breast tumors by simultaneously enhancing both HIF1alpha- and AP-1-mediated VEGFa transcription. Oncotarget.

[36] Peng M., Zhao G., Yang F., Cheng G., Huang J., Qin X., Liu Y., Wang Q., Li Y., Qin D. (2017). NCOA1 is a novel susceptibility gene for multiple myeloma in the Chinese population: A case-control study. PLoS ONE.

[37] Goebel E.A., Hernandez Bonilla S., Dong F., Dickson B.C., Hoang L.N., Hardisson D., Lacambra M.D., Lu F.I., Fletcher C.D.M., Crum C.P. (2020). Uterine Tumor Resembling Ovarian Sex Cord Tumor (UTROSCT): A Morphologic and Molecular Study of 26 Cases Confirms Recurrent NCOA1-3 Rearrangement. Am. J. Surg. Pathol..

[38] Walsh C.A., Bolger J.C., Byrne C., Cocchiglia S., Hao Y., Fagan A., Qin L., Cahalin A., McCartan D., McIlroy M. (2014). Global gene repression by the steroid receptor coactivator SRC-1 promotes oncogenesis. Cancer Res..

[39] Luo C., Zhou M., Chen C., Li S., Li Q., Huang Y., Zhou Z. (2022). A liposome-based combination strategy using doxorubicin and a PI3K inhibitor efficiently inhibits pre-metastatic initiation by acting on both tumor cells and tumor-associated macrophages. Nanoscale.

[40] Furuyama K., Kaneko K., Vargas P.D. (2007). Heme as a magnificent molecule with multiple missions: Heme determines its own fate and governs cellular homeostasis. Tohoku J. Exp. Med..

[41] Mao D., Reuter C.M., Ruzhnikov M.R.Z., Beck A.E., Farrow E.G., Emrick L.T., Rosenfeld J.A., Mackenzie K.M., Robak L., Wheeler M.T. (2020). De novo EIF2AK1 and EIF2AK2 Variants Are Associated with Developmental Delay, Leukoencephalopathy, and Neurologic Decompensation. Am. J. Hum. Genet..

[42] Grevet J.D., Lan X., Hamagami N., Edwards C.R., Sankaranarayanan L., Ji X., Bhardwaj S.K., Face C.J., Posocco D.F., Abdulmalik O. (2018). Domain-focused CRISPR screen identifies HRI as a fetal hemoglobin regulator in human erythroid cells. Science.

[43] Bastola P., Leiserowitz G.S., Chien J. (2022). Multiple Components of Protein Homeostasis Pathway Can Be Targeted to Produce Drug Synergies with VCP Inhibitors in Ovarian Cancer. Cancers.

[44] Liu Y.Y., Liu Y.K., Hu W.T., Tang L.L., Sheng Y.R., Wei C.Y., Li M.Q., Zhu X.Y. (2019). Elevated heme impairs macrophage phagocytosis in endometriosis. Reproduction.

